# KRT8 phosphorylation regulates the epithelial‐mesenchymal transition in retinal pigment epithelial cells through autophagy modulation

**DOI:** 10.1111/jcmm.14998

**Published:** 2020-02-05

**Authors:** Qi Miao, Yufeng Xu, Houfa Yin, Huina Zhang, Juan Ye

**Affiliations:** ^1^ Eye Center Second Affiliated Hospital of Zhejiang University School of Medicine Hangzhou China

**Keywords:** autophagy, epithelial‐mesenchymal transition, keratin 8, proliferative vitreoretinopathy, retinal pigment epithelial cells

## Abstract

Proliferative vitreoretinopathy (PVR) is a severe ocular disease which results in complex retinal detachment and irreversible vision loss. The epithelial‐mesenchymal transition (EMT) of retinal pigment epithelial (RPE) cells is considered to be critical in the pathogenesis of PVR. In this study, we focused on the potential impact of keratin 8 (KRT8) phosphorylation and autophagy on TGF‐β2–induced EMT of RPE cells and explored the relationship between them. Using immunofluorescence and Western blot analysis, the co‐localization of KRT8 and autophagy marker, as well as the abundance of phosphorylated KRT8 (p‐KRT8) expression, was observed within subretinal and epiretinal membranes from PVR patients. Moreover, during TGF‐β2–induced EMT process, we found that p‐KRT8 was enhanced in RPE cells, which accompanied by an increase in autophagic flux. Inhibition of autophagy with pharmacological inhibitors or specific siRNAs was associated with a reduction in cell migration and the synthesis of several EMT markers. In the meantime, we demonstrated that p‐KRT8 was correlated with the autophagy progression during the EMT of RPE cells. Knockdown the expression or mutagenesis of the critical phosphorylated site of KRT8 would induce autophagy impairment, through affecting the fusion of autophagosomes and lysosomes. Therefore, this study may provide a new insight into the pathogenesis of PVR and suggests the potential therapeutic value of p‐KRT8 in the prevention and treatment of PVR.

## 1 INTRODUCTION

Proliferative vitreoretinopathy (PVR) is a serious complication related to a number of intraocular diseases, including retinal detachment (RD) and ocular trauma. Moreover, PVR is the most common cause of surgical failure of rhegmatogenous RD (RRD).^1^ The characteristic of PVR is the formation of fibrotic membranes within vitreous cavity, subretina and retinal surface, which results in tractional retinal detachment and prevents the reattachment of the retina.^2^ Although the precise mechanism of PVR has not been completely clarified, retinal pigment epithelial (RPE) cells are considered to play a critical role in fibrotic membrane formation, as they undergo the epithelial‐mesenchymal transition (EMT) process.^3^ In this process, RPE cells lose their polarity and cell‐cell contact, trans‐differentiate into fibroblast‐like cells and produce extracellular matrix (ECM) components.^4^, ^5^ Previous studies have indicated that the EMT process of RPE cells can be triggered by various growth factors and cytokines, such as platelet‐derived growth factor (PDGF), transforming growth factor beta (TGF‐β), epidermal growth factor and tumour necrosis factor‐α.^6^ Among these, TGF‐β signalling molecule is involved in the EMT process of a variety of cell types and is considered the major regulator of EMT.^7^, ^8^

KRT8 (keratin 8) is one of the most important keratin proteins and considered as an epithelial marker. Besides maintaining the mechanical integrity of cells, KRT8 with its filament partner KRT18 have been shown to modulate cellular response to stress stimuli and contributed to cell resistance to apoptosis.^9^, ^10^ In addition, KRT8 has been reported to be involved in the EMT of gastric cancer cells.^11^ In recent studies, KRT8 was found to be elevated in the aqueous humour of age‐related macular degeneration (AMD) patients.^12^ In vitro studies also indicated that high KRT8 could protect RPE cells from degeneration under oxidative stress.^13^ However, the molecular mechanism of KRT8 in the pathogenesis of PVR is still poorly understood.

Autophagy is an evolutionary conserved process by which lysosomes degrade and recycle cytoplasmic materials including long‐lived proteins and old or damaged organelles.^14^ Under physiological conditions, autophagy is responsible for maintaining a proper cellular homeostasis. Alternatively, autophagy may be associated with apoptosis, tumorigenesis, cellular infection or ageing in response to numerous pathological stimuli.^15^, ^16^ In RPE cells, autophagy contributes to the energy balance and cellular quality control.^17^, ^18^ On the contrary, autophagy can provide possibility of cellular self‐destruction and result in cell death under chronic stress conditions.^19^, ^20^ Accumulating evidences have demonstrated that autophagy may participate in the fibrotic process of several human tissues.^21^, ^22^ Besides, recent observations have indicated that the autophagolysosomal pathway contributes to the EMT process of RPE cells.^23^, ^24^ Therefore, we have suggested that autophagy may be involved in the pathogenesis of PVR.

In this work, we have studied the autophagic response of RPE cells undergoing the EMT process induced by TGF‐β2, as well as the phosphorylation of KRT8, to explore the possible molecular mechanism in the development of PVR.

## 2 MATERIALS AND METHODS

### 2.1 Ethics statement

The study involving human participants was conducted according to the tenets of the Declaration of Helsinki and approved by the ethics committee of 2nd Affiliated Hospital, Medical College of Zhejiang University, Hangzhou, China. Appropriate informed consent was obtained from all participants.

### 2.2 Reagents and antibodies

Recombinant human TGF‐β2, 3‐methyladenine (3‐MA) and bafilomycin A1 (Baf‐A1) were purchased from PeproTech and Sigma‐Aldrich, respectively. The GFP‐LC3B plasmid was obtained from Addgene (24920), and adenovirus encoding for mRFP‐GFP‐LC3 was obtained from Hanbio Biotechnology. Rabbit anti‐human LC3B, p62, Atg5‐12, Beclin 1 and GAPDH were from Cell Signaling. Mouse anti‐human α‐SMA was from Sigma‐Aldrich. Rabbit anti‐human fibronectin, collagen IV, p‐KRT8, mouse anti‐human KRT8 and LAMP2 were from Abcam. Goat anti‐rabbit or antimouse horseradish peroxidase (HRP)‐labelled secondary antibodies were from Thermo Fisher Scientific. Mouse IgG isotype control, rabbit IgG isotype control, AlexaFluor488‐conjugated donkey antimouse and AlexaFluor555‐conjugated donkey anti‐rabbit secondary antibodies were from Life Technologies.

### 2.3 Cell culture and treatment

Human retinal pigment epithelial cell line (ARPE‐19) was obtained from American type culture collection (ATCC) and cultured in DMEM/F12 (Gibco) medium supplemented with 10% foetal bovine serum (FBS; Gibco). Human primary RPE cells were isolated from post‐mortem donor eyes provided by the eye bank of Second Affiliated Hospital, Zhejiang University School of Medicine (Hangzhou, China) and cultured as previously described.^25^ ARPE‐19 and human primary RPE cells were maintained in a 37°C incubator with 5% CO_2._ For some experiments, equal number of cells were plated and cultivated in serum‐free medium for 12 hours before stimulation with 10 ng/mL TGF‐β2 for different durations. For 3‐MA or Baf‐A1 treatment, the cells were pre‐treated with 3‐MA (10 mmol/L) or Baf‐A1 (10 nmol/L) for 4 hours prior to the addition of TGF‐β2 for various time‐points.

### 2.4 Construction of plasmids which encode wild‐type KRT8 or KRT8‐S74A

The coding sequence of wild‐type human KRT8 was amplified by PCR with the following primers: sense primer 5′‐TCGAGAATTCTGCCTCTACCATGTCCATC‐3′; antisense primer 5′‐ TCGAGATATCTGTTCCCAGTGCTACCCT‐3′. The PCR products were gel purified, double digested with EcoRI and EcoRV and then cloned into a eukaryotic expression vector, pcDNA3.1+ (Invitrogen). The plasmid expressing KRT8‐S74A was obtained by site‐directed mutagenesis with the following primers: sense primer 5′‐ CAACCAGAGCCTGCTGGCCCCCCTTGTCCTGGAG‐3′; antisense primer 5′‐ CTCCAGGACAAGGGGGGCCAGCAGGCTCTGGTTG‐3′. Both wild‐type and mutant constructs were confirmed through directly sequencing.

### 2.5 RNA interference

RNA interference was achieved by transfection of siRNA with Lipofectamine 3000 reagents (Invitrogen) according to the manufacturer's instruction. The siRNAs were used at 50 nmol/L, and the sequences were as follows: si‐ATG5 (5′‐GGATGAGATAACTGAAAGG‐3′); si‐BECN1 (5′‐CAGUUUGGCACAAUCAAUA‐3′); si‐KRT8 (5′‐CUGAGAUGAACCGGAACAU‐3′); negative control (NC) siRNA (5′‐UUCUCCGAACGUGUCACGU‐3′). All RNA oligonucleotides were synthesized by GenePharm.

### 2.6 Wound healing assays

Cells were seeded in 6‐well plates and grown to 90% confluence after siRNAs transfection. Then, the cells were wounded by scraping them with a sterile plastic tip and cultured in FBS‐free DMEM/F12 with or without TGF‐β2 for 48 hours. To analyse the cell migration, the wounded areas were photographed under a phase‐contrast microscope (Olympus) and processed by ImageJ analysis software (NIH). Percentage of wound healing was calculated using following equation:$\left[1-\left(\text{empty}\;\text{area}\;X\;h/\text{empty}\;\text{area}\;0\;h\right)\right]\times 100$.

### 2.7 GFP‐LC3B fluorescence analysis

Cells were seeded in 60‐mm dishes and transfected with GFP‐LC3B using Lipofectamine 3000. After the indicated treatments, cells were fixed with 4% paraformaldehyde. Nuclei were stained with Hoechst33258 (Sigma‐Aldrich). Specimens were observed, and images were captured using Olympus FluoView 1000 confocal microscope (Olympus).

### 2.8 Autophagosome fusion assay

Cells were seeded on cover slips in 24‐well plates and infected with the adenovirus encoding for mRFP‐GFP‐LC3 24 hours prior to the stimulation with TGF‐β2. After the indicated time, cells were fixed with 4% paraformaldehyde and observed for GFP fluorescence and mRFP fluorescence under an Olympus FluoView 1000 confocal microscope (Olympus).

### 2.9 Immunofluorescence staining

PVR membrane specimens were fixed in 4% paraformaldehyde solution and washed with PBS. Then, the samples were permeabilized with 0.1% Triton‐X‐100 (Sigma‐Aldrich) and blocked with 2.5% BSA in PBS. Subsequently, all samples were immunostained with the indicated primary antibodies overnight at 4°C and the indicated secondary antibodies for 2 hours at room temperature. Thereafter, samples were counterstained with DAPI (Sigma‐Aldrich) and examined with an Olympus FluoView 1000 confocal microscope (Olympus).

For cellular immunofluorescence staining, cells were fixed in 4% paraformaldehyde solution for 15 minutes, permeabilized with 0.1% Triton‐X‐100 (Sigma‐Aldrich) for 15 minutes and then blocked with 2.5% BSA for 1 hour. In the following, cells were incubated with the indicated primary antibodies overnight at 4°C and the indicated secondary antibodies for 1 hour at room temperature. After being washed, cells were observed with an Olympus FluoView 1000 confocal microscope (Olympus).

### 2.10 Western blot analysis

Human tissues or culture cells were lysed in RIPA buffer (Beyotime Biotechnology). The protein concentration was determined by a BCA kit (Thermo). Same amounts of protein were separated by electrophoresis in 10%‐15% SDS‐PAGE and subsequently transferred to nitrocellulose membranes. Blots were blocked with 5% bovine serum albumin (BSA) and then incubated with primary antibodies overnight at 4°C. Blots were subsequently incubated with HRP‐conjugated secondary antibodies for 1 hour at room temperature, and bound antibodies were visualized with ECL on X‐ray film. The intensities of the bands were measured by Quantity One (Bio‐Rad) and normalized to each corresponding loading control GAPDH.

### 2.11 Human PVR membranes and retinal tissues collection

PVR membrane specimens were acquired from patients undergoing standard vitreoretinal surgery for the treatment of retinal detachment complicated with PVR. Retinal tissues were obtained from the normal donor eyes from the eye bank of Second Affiliated Hospital, Zhejiang University School of Medicine (Hangzhou, China). All the samples were stored at −80°C immediately after extraction until the experiments were conducted.

### 2.12 Statistical analysis

Results were expressed as means ± SEM Data analysis was performed using GraphPad Prism 6.0 software (GraphPad Software). For comparison of two groups, statistical difference was determined by Student's *t* test. A one‐way ANOVA followed by Tukey *post hoc* test was used for multiple comparisons. A value of *P* < .05 was considered statistically significant.

## 3 RESULTS

### 3.1 Expression of KRT8 and its phosphorylated form, and autophagy marker within PVR membranes

**Figure 1:**
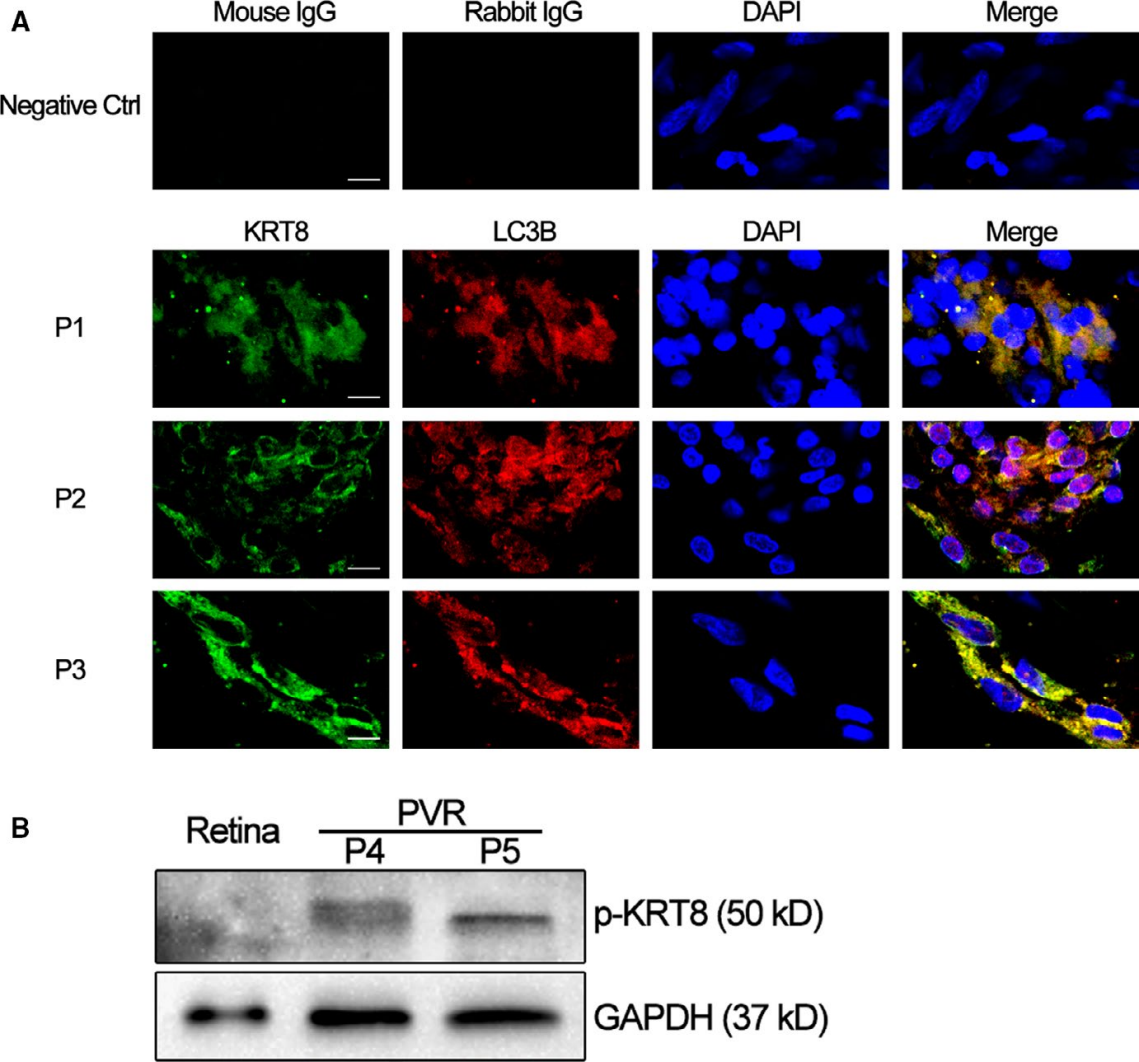
Expression of KRT8 and its phosphorylated form, and autophagy marker in human PVR membranes. A, Representative fluorescence microscopy images show the distributions of immunoreactive KRT8 (green fluorescence) and LC3B (red fluorescence) within the subretinal and epiretinal membranes from three independent PVR patients. Yellow or orange fluorescence resulted from the overlay of green and red fluorescence, which indicates the co‐localization of KRT8 with LC3B. Nuclei were stained with DAPI and are represented with blue fluorescence. The upper panel shows the representative immunofluorescence staining of negative control using mouse and rabbit control IgG. Scale bar = 10 µm. B, Western blot analysis of p‐KRT8 in the retina from normal donor eye and subretinal and epiretinal membranes from two independent PVR patients. GAPDH levels were used as loading control

To investigate whether KRT8 and autophagy are involved in the pathogenesis of PVR, we first examined the expression of KRT8 and LC3B by immunofluorescence within the subretinal and epiretinal membranes from three independent patients with PVR. The characteristics of the patients are summarized in Table 1, and the statuses of their fundus are shown in Figure S1. As shown in Figure 1A, dense KRT8 and LC3B fluorescence were present within the subretinal and epiretinal membranes, and the co‐localization of KRT8 and LC3B was also observed. Moreover, immunofluorescence with mouse and rabbit control IgG (Negative Ctrl) using the same tissues did not show any specific staining, which enhanced the anti‐KRT8 and anti‐LC3B staining specificity. Besides, we also examined the phosphorylated form of KRT8 (p‐KRT8) expression by Western blot using subretinal and epiretinal membranes from two independent patients with PVR (Table 1). Compared with retinal tissues from the normal donor eye, the abundance of p‐KRT8 expression was observed in both subretinal and epiretinal membranes (Figure 1B). As RPE cells are the only epithelial cells in proliferative membranes,^26^ it is expected that the crosstalk between KRT8/p‐KRT8 and autophagy in RPE cells contributes to the pathogenesis of PVR.

**Table 1:** Characteristics of the patients for immunofluorescence staining and Western blot analysis

Patient No.	Age (y)	Sex	Tissues	Applications
P1	53	Female	Subretinal membrane	IF
P2	71	Male	Epiretinal membrane	IF
P3	28	Female	Subretinal membrane	IF
P4	48	Male	Subretinal membrane	WB
P5	49	Female	Epiretinal membrane	WB

Abbreviations: IF, immunofluorescence; WB, Western blot.

### 3.2 TGF‐β2 simultaneously induces phosphorylation of KRT8 and autophagy in RPE cells

**Figure 2:**
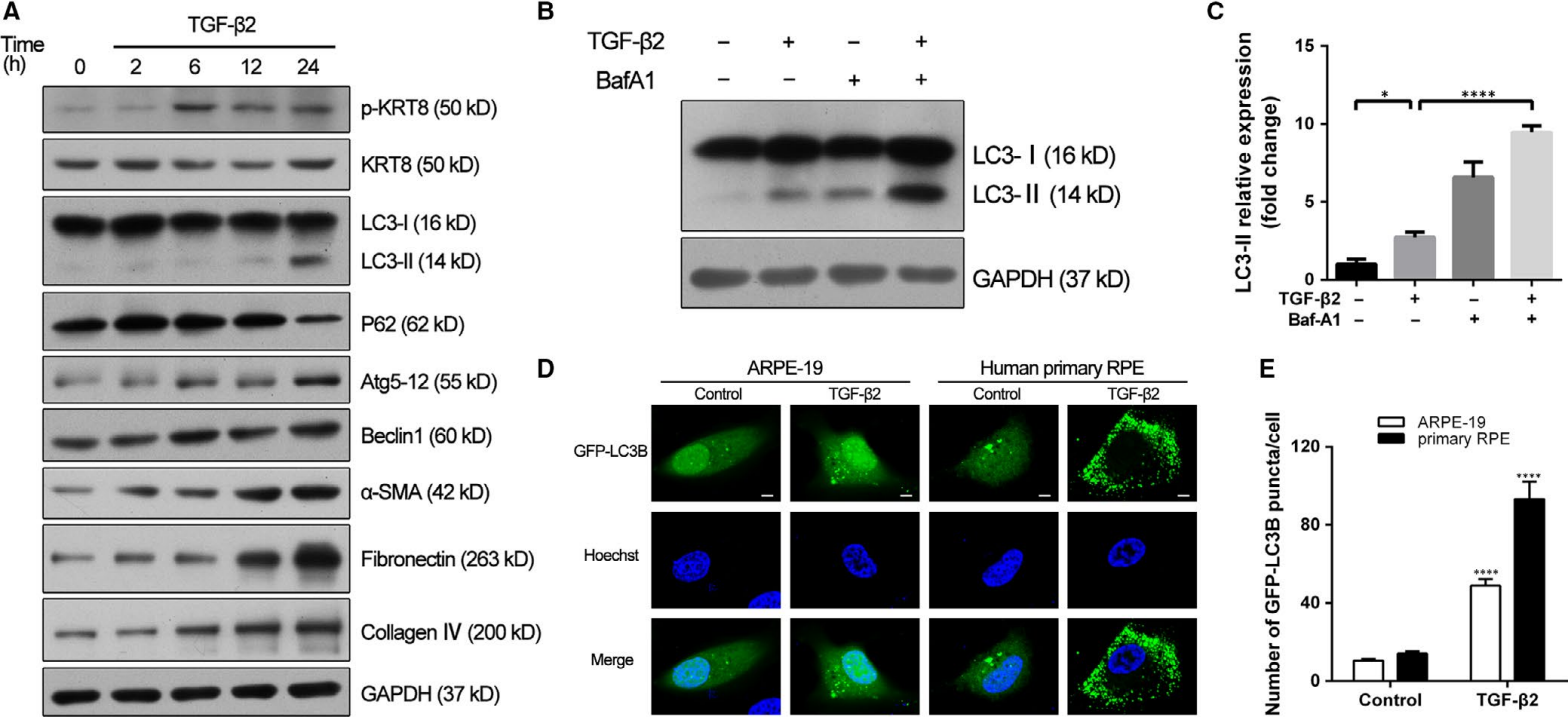
KRT8 phosphorylation and autophagic flux are increased during TGF‐β2–induced EMT in RPE cells. A, Western blot analysis of p‐KRT8, KRT8, autophagy hallmark proteins (LC3‐II, p62, Atg5‐12 and Beclin 1) and EMT markers (α‐SMA, fibronectin and collagen IV) in ARPE‐19 cells treated with TGF‐β2 (10 ng/mL) for the indicated hours. GAPDH levels were used as loading control. B, Western blot analysis of LC3‐II in ARPE‐19 cells treated with TGF‐β2 (10 ng/mL, 24 h) either in the presence or absence of the autophagy inhibitor Baf‐A1 (10 nmol/L). GAPDH was used as loading control. C, Bar graph shows the relative expression level of LC3‐II (normalized to GAPDH) in Western blot analysis. The data are presented as mean ± SEM, n = three independent experiments. *P < .05 and ****P < .0001. D, The localization of GFP‐LC3B puncta in ARPE‐19 and human primary RPE cells treated with TGF‐β2 (10 ng/mL) for 24 h. Nuclei were stained with Hoechst33258 and are represented with blue fluorescence. Scale bar = 5 µm. E, Bar graph indicates the average number of LC3B puncta per cell obtained in fluorescence analysis. The data are presented as mean ± SEM, n = 20. ****P < .0001

To mimic the EMT process of RPE cells, we used TGF‐β2 which is the predominant TGF‐β isoform in the posterior eye,^27^ as the inducer of EMT. When ARPE‐19 cells were treated with TGF‐β2 (10 ng/mL) for various time periods, the EMT markers such as α‐smooth muscle actin (α‐SMA), fibronectin (FN) and collagen IV (Col IV) showed a time‐dependent up‐regulation, suggesting RPE cells were undergoing the EMT process. Meantime, while the expression of KRT8 did not change significantly, the phosphorylated form (p‐KRT8) was enhanced during the EMT process. In addition, increased expression of LC3‐II, Atg5‐12 and Beclin 1, as well as the gradual degradation of p62, was also observed in RPE cells (Figure 2A). Moreover, the accumulation of LC3‐II was further enhanced in the presence of bafilomycin A1 (Baf‐A1), which inhibits the fusion of autophagosomes and lysosomes (Figure 2B,C). These results indicated that autophagy was activated and the autophagic flux was increased in RPE cells during EMT. The autophagic response of RPE cells during EMT was further confirmed by GFP‐LC3B puncta assay, in which the average number of GFP‐LC3B puncta per cell in both ARPE‐19 and human primary RPE cells dramatically increased by approximately 5‐ to 6‐fold, compared with the control cells (Figure 2D,E).

### 3.3 Suppression of autophagy inhibits the EMT process of RPE cells

**Figure 3:**
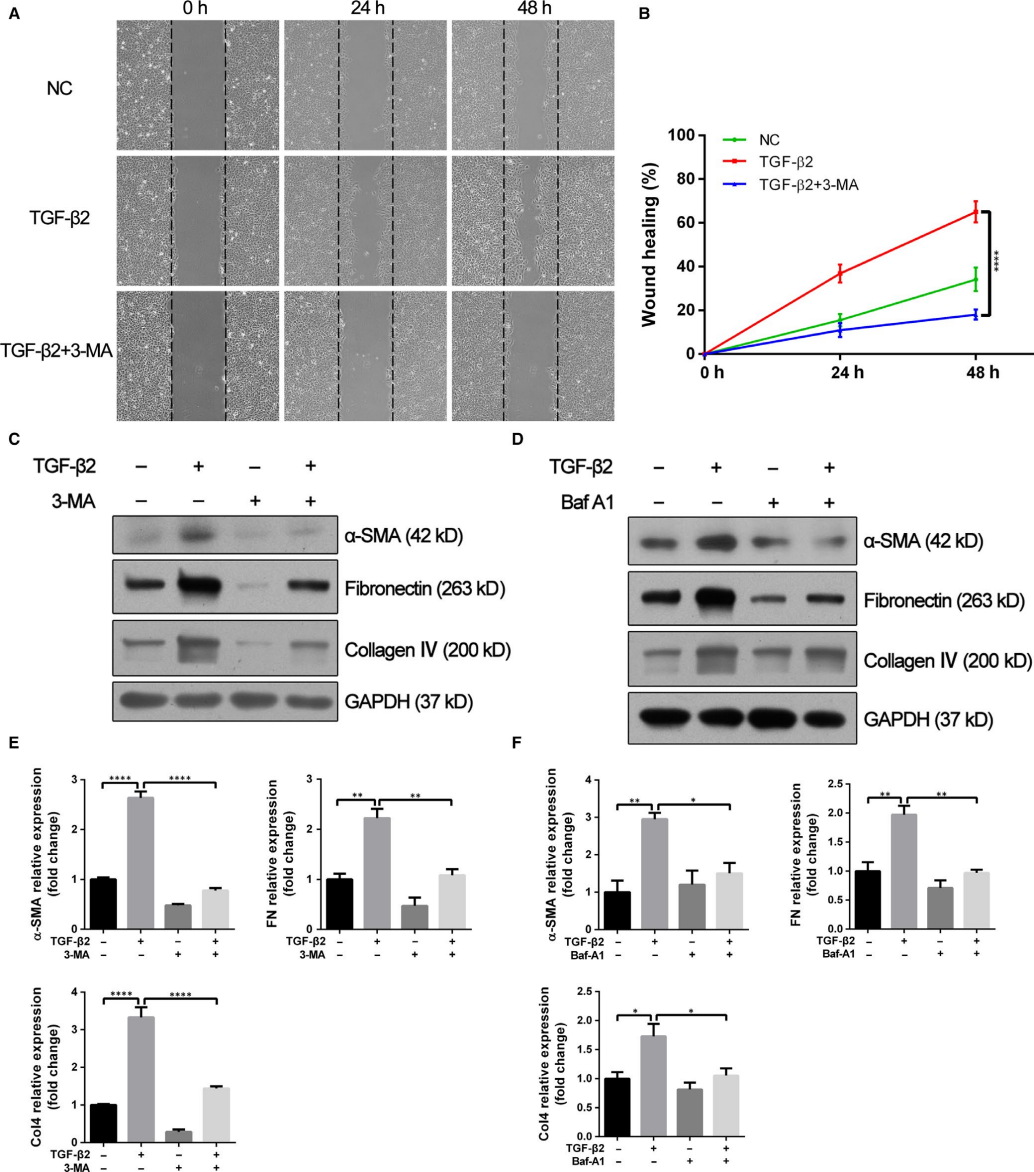
Autophagy inhibitors attenuate TGF‐β2–induced cell migration and EMT markers synthesis. A, Wound healing assays of TGF‐β2 (10 ng/mL) stimulated ARPE‐19 cells co‐treatment with or without 3‐MA (10 mmol/L). Phase‐contrast microphotographs (4 × objective) were acquired at 0, 24 and 48 h after scratching. B, Graph shows the percentage of wound healing area relative to 0 h. The data are presented as mean ± SEM, n = three independent experiments. ****P < .0001. C, D, Western blot analysis of α‐SMA, fibronectin and collagen IV in ARPE‐19 cells treated with TGF‐β2 (10 ng/mL) in the absence or presence of either 3‐MA (10 mmol/L) or Baf‐A1 (10 nmol/L) for 24 h. GAPDH was used as loading control. E, F, Bar graphs indicate the relative expression levels of α‐SMA, fibronectin and collagen IV (normalized to GAPDH) in Western blot analysis. The data are presented as mean ± SEM, n = three independent experiments. *P < .05, **P < .01 and ****P < .0001

**Figure 4:**
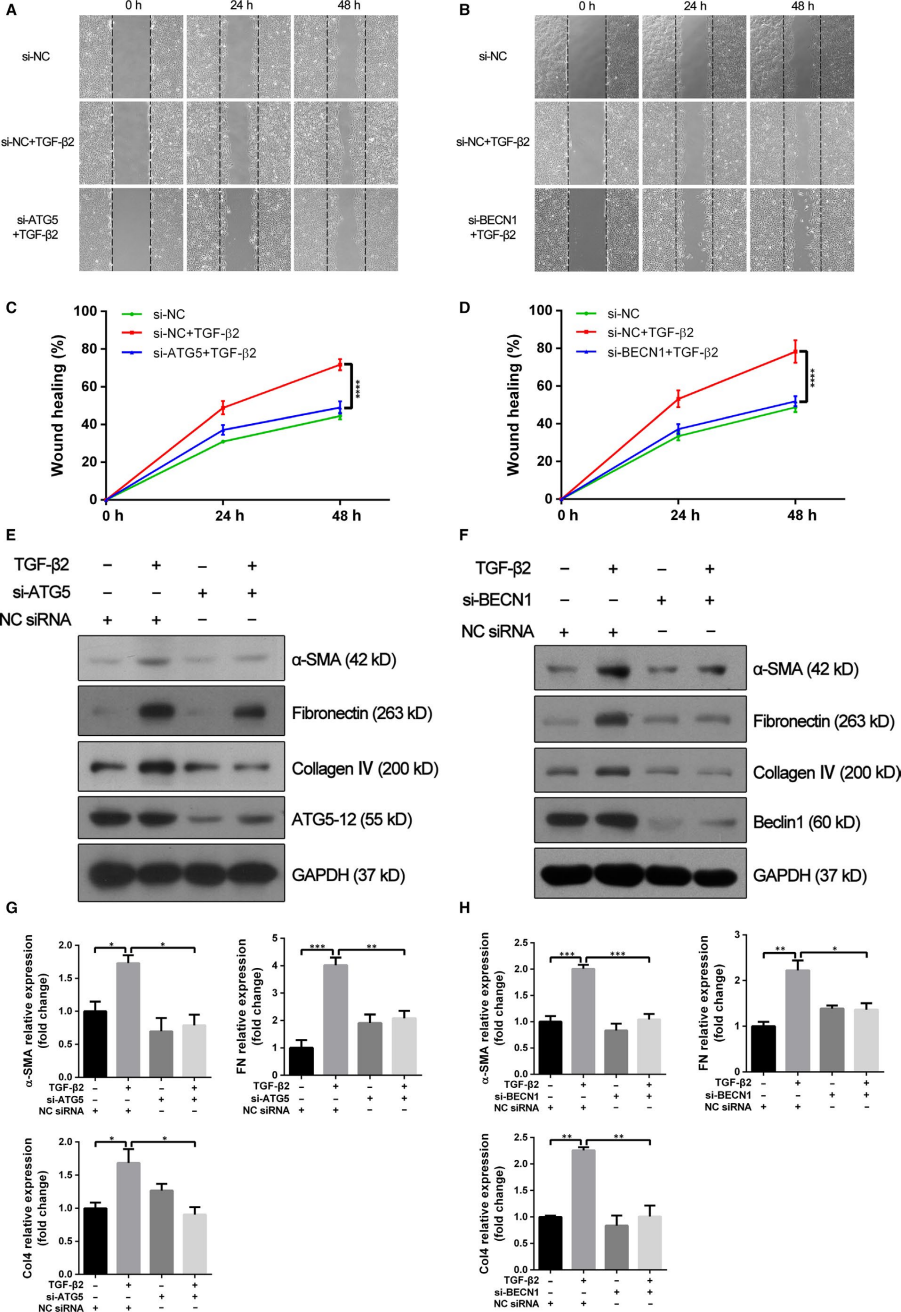
Knockdown of ATG5 or Beclin 1 attenuates enhanced cell migration and EMT markers synthesis induced by TGF‐β2. A, B, Wound healing assays were performed on ATG5‐ and Beclin 1‐depleted ARPE‐19 cells (si‐ATG5 and si‐BECN1, respectively) and on control cells (NC siRNA) with or without TGF‐β2 (10 ng/mL) treatment. Phase‐contrast microphotographs (4 × objective) were obtained at 0, 24 and 48 h after scratching. C, D, Graph shows the relative wound healing area normalized to 0 h. The data are presented as mean ± SEM, n = three independent experiments. ****P < .0001. E, F, Western blot analysis of α‐SMA, fibronectin, collagen IV, Atg5‐12 and Beclin 1 in NC siRNA, si‐ATG5 or si‐BECN1 transfected ARPE‐19 cells with or without TGF‐β2 (10 ng/mL) treatment for 24 h. GAPDH was used as loading control. G, H, Bar graphs indicate the relative expression level of each protein (normalized to GAPDH) in Western blot analysis. The data are presented as mean ± SEM, n = three independent experiments. *P < .05, **P < .01 and ***P < .001

To investigate the role of autophagy activity in TGF‐β2–induced EMT, two pharmacological inhibitors of autophagy, 3‐methyladenine (3‐MA) and Baf‐A1 were used. In wound healing assay, while ARPE‐19 cells stimulated by TGF‐β2 exhibited an enhanced ability of migration and repaired the wound much faster, co‐treatment with 3‐MA led to retarded wound healing (Figure 3A,B). Moreover, the increased expression levels of mesenchymal specific proteins such as α‐SMA, fibronectin and collagen IV induced by TGF‐β2 were significantly abrogated by co‐treatment with 3‐MA or Baf‐A1 in both ARPE‐19 and human primary RPE cells (Figure 3C‐F and Figure S2A,B). To utilize a non‐pharmacological approach to confirm the results obtained with pharmacological autophagy inhibitors, the expression of ATG5 and Beclin1 (BECN1), two fundamental genes for autophagy induction, was silenced by specific siRNA in ARPE‐19 cells. Similar to above results, the lack of either ATG5 or BECN1 affected significantly TGF‐β2–induced cell migration (Figure 4A‐D) and resulted in decreased expression levels of α‐SMA, fibronectin and collagen IV, when compared with cells transfected with the negative control siRNA (Figure 4E‐H). These findings demonstrated that suppression of autophagy could inhibit the TGF‐β2 mediated EMT process in RPE cells.

### 3.4 Phosphorylation of KRT8 correlates with autophagy progression during EMT

**Figure 5:**
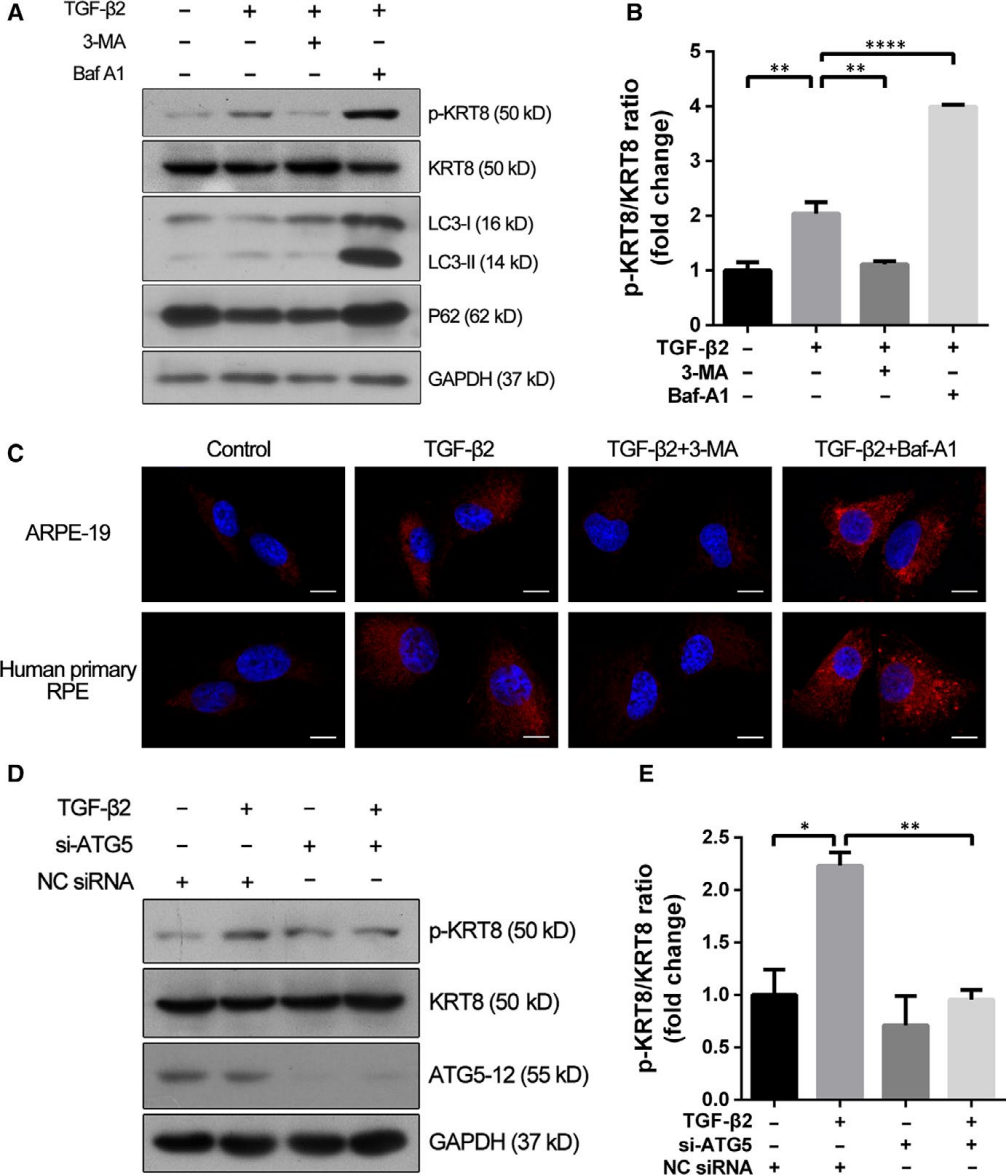
KRT8 phosphorylation is correlated with autophagy during TGF‐β2 mediated EMT in RPE cells. A, Western blot analysis of p‐KRT8, KRT8, LC3‐II and p62 present in ARPE‐19 cells exposed to TGF‐β2 (10 ng/mL) in the absence or presence of either 3‐MA (10 mmol/L) or Baf‐A1 (10 nmol/L) for 24 h. GAPDH was used as loading control. B, Bar graph shows the p‐KRT8:KRT8 ratio in Western blot analysis. The data are presented as mean ± SEM, n = three independent experiments. **P < .01 and ****P < .0001. C, Representative fluorescence microscopy images show the expression of p‐KRT8 (red fluorescence) in ARPE‐19 and human primary RPE cells. Cells were exposed to TGF‐β2 (10 ng/mL) in the absence or presence of either 3‐MA (10 mmol/L) or Baf‐A1 (10 nmol/L) for 24 h. Nuclei were stained with DAPI and are represented with blue fluorescence. Scale bar = 10 µm. D, Western blot analysis of p‐KRT8, KRT8 and Atg5‐12 in NC siRNA or si‐ATG5 transfected ARPE‐19 cells treated with or without TGF‐β2 (10 ng/mL) for 24 h. GAPDH was used as loading control. E, Bar graph shows the p‐KRT8:KRT8 ratio in Western blot analysis. The data are presented as mean ± SEM, n = three independent experiments. *P < .05 and **P < .01

To determine the relationship between KRT8 and the autophagy induced by TGF‐β2, we first evaluated the effect of autophagy inhibition on the expression and its phosphorylated form of KRT8 (Figure 5A,B). As expected, the expression of KRT8 did not change significantly when ARPE‐19 cells co‐treated with 3‐MA or Baf‐A1. However, these cells showed decreased expression of p‐KRT8 after the initial stage of autophagy has been blocked by 3‐MA, as indicated by reduced LC3‐II conversion and p62 degradation. In contrast, inhibition of the late stage of autophagy by Baf‐A1 (accumulation of LC3‐II and p62) further enhanced the up‐regulation of p‐KRT8 induced by TGF‐β2. These results were consistent with the expression pattern of p‐KRT8 in both ARPE‐19 and human primary RPE cells observed through immunofluorescence staining (Figure 5C). Moreover, the increased expression of p‐KRT8 caused by TGF‐β2 could also be attenuated by knockdown of ATG5, which resulted in the inactivation of autophagy initiation (Figure 5D,E). These results indicated that the KRT8 phosphorylation was related to the progression of autophagy during EMT process.

### 3.5 Phosphorylation of KRT8 at Ser74 is essential for autophagosome‐lysosome fusion in RPE cells

**Figure 6:**
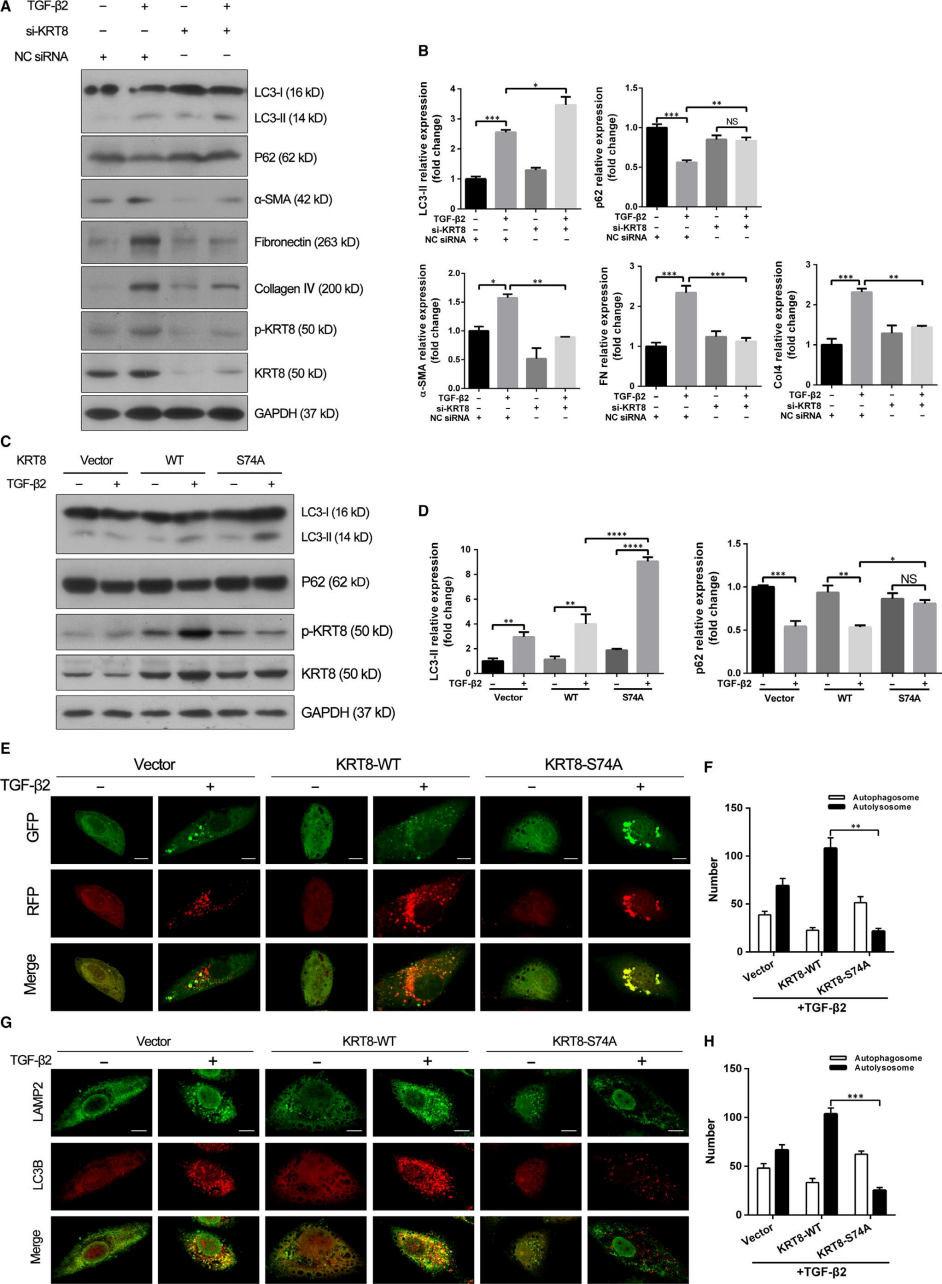
Phosphorylated KRT8 is critical to autophagosome‐lysosome fusion in RPE cells. A, Western blot analysis of p‐KRT8, KRT8, LC3‐II, p62 and EMT markers (α‐SMA, fibronectin, collagen IV) in NC siRNA or si‐KRT8 transfected ARPE‐19 cells treated with or without TGF‐β2 (10 ng/mL) for 24 h. GAPDH was used as loading control. B, Bar graphs indicate the relative expression of each protein (normalized to GAPDH) in Western blot analysis. The data are presented as mean ± SEM, n = three independent experiments. NS represents no significance, *P < .05, **P < .01 and ***P < .001. C, Western blot analysis of p‐KRT8, KRT8, LC3‐II and p62 in ARPE‐19 cells transfected with empty vector, wild‐type KRT8 or KRT8‐S74A mutant with or without TGF‐β2 (10 ng/mL, 24 h) treatment. GAPDH was used as loading control. D, Bar graphs show the relative expression of each protein (normalized to GAPDH) in Western blot analysis. The data are presented as mean ± SEM, n = three independent experiments. NS represents no significance, *P < .05, **P < .01, ***P < .001 and ****P < .0001. E, Representative fluorescence microscopy images of autophagosomes (yellow puncta) and autolysosomes (red puncta) in the Ad‐mRFP‐GFP‐LC3 infected ARPE‐19 cells transfected with empty vector, wild‐type KRT8 or KRT8‐S74A mutant with or without TGF‐β2 (10 ng/mL, 24 h) stimulation. Scale bar = 10 µm. F, Bar graph indicates the average number of autophagosomes and autolysosomes in each cell obtained in fluorescence analysis. The data are presented as mean ± SEM, n = three independent experiments. **P < .01. G, Representative fluorescence microscopy images of LC3B‐labelled autophagosomes (red fluorescence) and LAMP2‐labelled lysosomes (green fluorescence) in ARPE‐19 cells transfected with empty vector, wild‐type KRT8 or KRT8‐S74A mutant with or without TGF‐β2 (10 ng/mL, 24 h) treatment. Scale bar = 10 µm. H, Bar graph indicates the average number of autophagosomes and autolysosomes in each cell obtained in fluorescence analysis. The data are presented as mean ± SEM, n = three independent experiments. ***P < .001

To examine the role of KRT8 and its phosphorylation in autophagic process under TGF‐β2 stimulation, the specific siRNA targeting KRT8 was used. As shown in Figure 6A, the si‐KRT8 successfully suppressed the expression of KRT8 and its phosphorylated form in ARPE‐19 cells. Moreover, under TGF‐β2 stimulation, the KRT8 knockdown resulted in increased LC3‐II conversion and decreased p62 degradation, as well as decreased expression of EMT markers such as α‐SMA, fibronectin and collagen IV (Figure 6A,B).

To confirm the effect of p‐KRT8 on autophagic process, ARPE‐19 cells were transfected with empty vector, wild‐type KRT8 and KRT8‐S74A mutant, respectively (Figure 6C,D). Cells transfected with wild‐type KRT8 or KRT8‐S74A mutant significantly increased the expression of KRT8. However, the increased expression of p‐KRT8 caused by TGF‐β2 was attenuated in cells transfected with KRT8‐S74A mutant. Meanwhile, cells expressing KRT8‐S74A showed increased LC3‐II and p62 levels, which suggested the inhibition of autophagic flux. These results were further confirmed by the autophagosome fusion assay, in which ARPE‐19 cells were transfected with mRFP‐GFP‐LC3 to label autophagosomes (yellow). When autophagosomes fuse with lysosomes to form autolysosomes, the GFP fluorescent signal is quenched and only mRFP is visible. Under TGF‐β2 stimulation, cells expressing wild‐type KRT8 showed an increased number of red puncta. In contrast, only large amounts of yellow puncta were observed in cells expressing KRT8‐S74A, which indicated the impairment of late stage of autophagy (Figure 6E,F). Therefore, the effect of KRT8‐S74A on fusion between autophagosomes and lysosomes was investigated by double labelling ARPE‐19 cells with LC3B (an autophagosome marker) and LAMP‐2 (a lysosome marker). Similar to the above result, wild‐type KRT8‐overexpressing cells treated with TGF‐β2 showed well co‐localization of LC3B‐positive autophagosomes and LAMP‐2‐positive lysosomes. By contrast, the co‐localization of LC3B and LAMP‐2 was dramatically reduced in cells expressing KRT8‐S74A (Figure 6G,H). These data indicated that phosphorylation of KRT8 at Ser74 was likely to facilitate the autophagosome‐lysosome fusion during TGF‐β2–induced autophagic process in RPE cells.

## 4 DISCUSSION

In the present study, we observed the expression pattern of KRT8 and its phosphorylated form, as well as LC3B in the surgically excised subretinal and epiretinal membranes, which directly represent the pathogenesis of PVR in vivo. Using an in vitro EMT model in which RPE cells were stimulated with TGF‐β2, we found that the phosphorylation of KRT8 and autophagic flux in RPE cells was increased during the EMT process. These results indicated that the crosstalk between phosphorylated KRT8 and autophagy was likely to be involved in the pathogenesis of PVR, which has not yet been elucidated.

Autophagy is a catabolic process that plays a fundamental role in maintaining the homeostasis of RPE cells. Autophagy can be activated as a cellular protective response when RPE cells are exposed to sustained oxidative stress. Impairment of autophagy is likely to induce an accumulation of damaged organelle and toxic proteins, which can lead to RPE cells dysfunction or death.^17^ In contrast, uncontrolled and excessive autophagy activation can drive RPE cells towards autophagy‐associated cell death.^20^ As autophagy is critical to the integrity and fate of RPE cells, the role of autophagy in the EMT process is also of concern. In gastrulation of chick embryos, inhibition of autophagy results in developmental delay by means of modulation of EMT process.^28^ Autophagy is also required for the fibrogenesis induced by TGF‐β1 in human atrial myofibroblasts.^29^ On the other hand, while autophagy supports viability of metastatic cancer cells during EMT,^30^ it also acts to prevent or reverse the EMT phenotype of some cancer cells.^31^, ^32^ These observations define a complex interaction between autophagy and EMT, which is influenced by several aspects. In this study, with the help of pharmacological autophagy inhibitors including 3‐MA and Baf‐A1, we have demonstrated that inhibition of autophagy significantly attenuates the TGF‐β2 mediated EMT of RPE cells, which is consistent with the results obtained by knockdowning the expression of ATG5 and Beclin1. These results support the hypothesis that TGF‐β2–induced EMT of RPE cells is mediated by autophagy.

KRT8, a well‐known epithelial marker protein, is a major component of keratin proteins. In epithelial cells, keratins can form structure networks linking the plasma membrane, nucleus and other cytoskeletal components and constitute the largest and most complex class of intermediate filaments (IFs).^33^, ^34^ Keratins are subdivided into type I (K9‐K20) and type II (K1‐K8) and are obligate heterodimers consisting of type I and II proteins in a 1:1 ratio.^35^ The type I and type II keratin monomers pair in certain combinations depending on a tissue or differentiation type.^36^ KRT8 has been known to pair with KRT18 to form the K8‐K18 heterodimer. Except for supporting the structural integrity of cells, accumulating evidences have shown that keratins are involved in the regulation of cell growth, cell migration and cellular response to stress‐related stimuli.^37^, ^38^, ^39^ As the structures of keratins are highly dynamic, phosphorylation targeting one or more Ser/Thr residues is needed for reorganization of keratins during some physiological or pathophysiological events.^40^, ^41^ Previous studies have demonstrated that phosphorylated KRT8 increases cell migration in epithelial cells.^42^, ^43^ Substitution of the major phosphorylation site in KRT8 (Ser74) or KRT18 (Ser53) increases apoptosis and liver injury in KRT8 transgenetic mice.^44^, ^45^ In this work, we found that the phosphorylated form rather than the expression of KRT8 correlates with the progression of autophagy during the EMT of RPE cells. The phosphorylation of KRT8 could be attenuated or enhanced when the initial or the late stage of autophagy was blocked, respectively. Moreover, knockdown the expression or alteration of the critical phosphorylation site (Ser74) of KRT8 induced autophagy impairment, through affecting the fusion of autophagosomes and lysosomes. These results were inconsistent with the recent studies, in which phosphorylated KRT8 is always in parallel with the total KRT8 expression, and the latter facilitates the autophagic process and plays a protective role in RPE cells under oxidative stress.^13^ As demonstrated by previous reports, phosphorylation of IFs can modulate their interaction with IF‐related proteins, such as adaptor protein 14‐3‐3.^46^, ^47^ 14‐3‐3 can link phosphorylated vimentin (one kind of IFs) with phosphorylated Beclin 1 to form a Beclin 1/14‐3‐3/vimentin complex, which promotes autophagy inhibition and tumorigenesis.^48^ Thus, further studies will be needed to identify the molecular mechanism responsible for autophagy regulation through the phosphorylation of KRT8.

In conclusion, this study provides evidence that KRT8 phosphorylation and autophagy are involved in the EMT of RPE cells. During this process, autophagy is activated and plays an indispensable role. Moreover, phosphorylated KRT8 is correlated with the progression of autophagy and contributes to the autophagosome‐lysosome fusion. Therefore, this will influence the process of EMT in RPE cells. Our findings provide a novel network for understanding the pathogenesis of PVR and highlight KRT8 phosphorylation as a putative novel therapeutic target for the prevention and treatment of human PVR.

## CONFLICT OF INTEREST

The authors declare that they have no conflict of interest.

## AUTHOR CONTRIBUTIONS

JY conceived the project, funded this study and revised the manuscript. QM designed and performed the experiments and wrote the manuscript. YFX and HFY performed statistical analysis. HNZ provided assistance in sample collection. All authors have read and approved the final manuscript.

## Data Availability

The data that support the findings of this study are available from the corresponding author upon reasonable request.
