# Supplementary figures and images for: KRT8 phosphorylation regulates the epithelial‐mesenchymal transition in retinal pigment epithelial cells through autophagy modulation
Source: J Cell Mol Med. 2020 Feb 5;24(5):3217–28. doi: 10.1111/jcmm.14998 (PMC7077598; doi:10.1111/jcmm.14998)

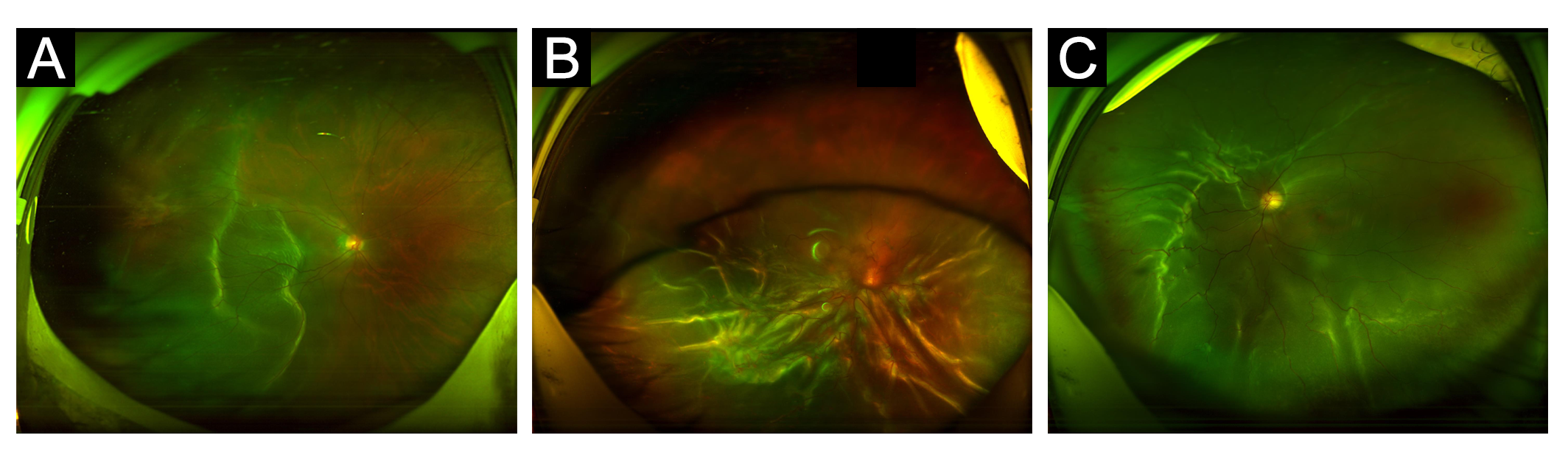

Supplement: Supplementary file 1 [file JCMM-24-3217-s001.tif]

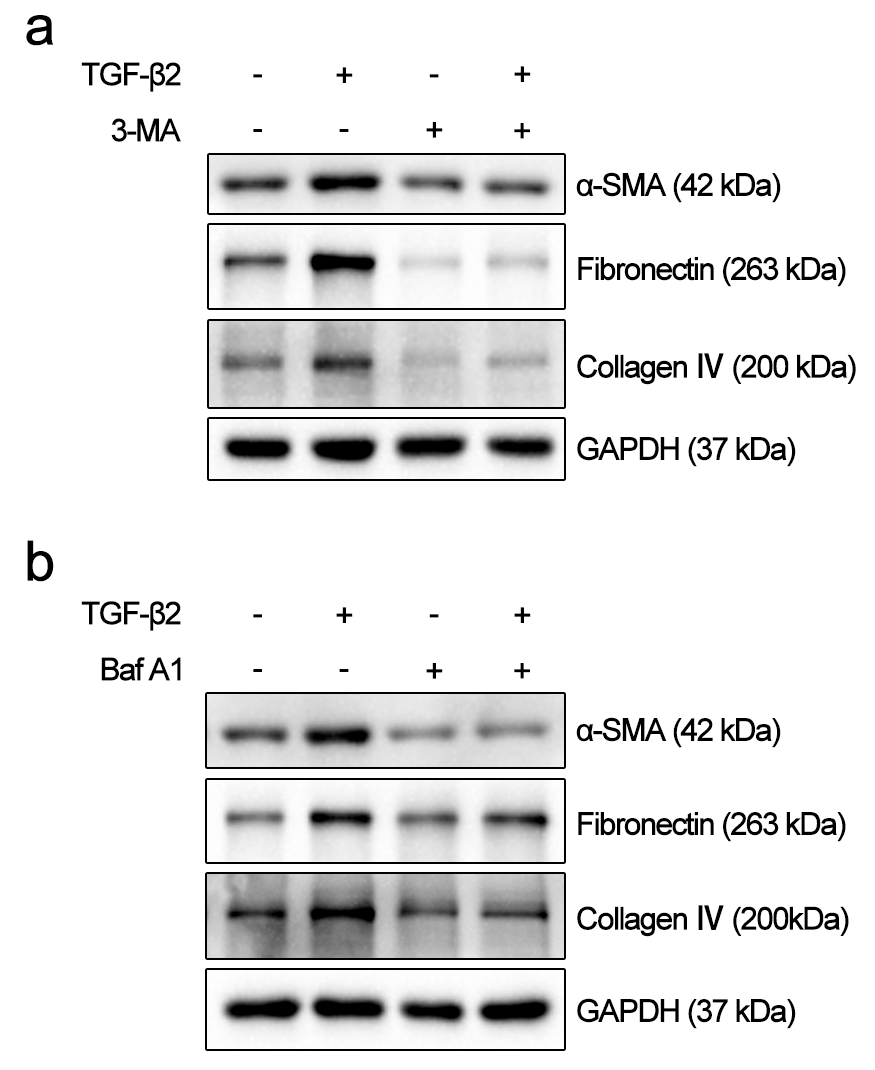

Supplement: Supplementary file 2 [file JCMM-24-3217-s002.tif]
