# Supplementary material for: KRT8 phosphorylation regulates the epithelial‐mesenchymal transition in retinal pigment epithelial cells through autophagy modulation
Source: J Cell Mol Med. 2020 Feb 5;24(5):3217–28. doi: 10.1111/jcmm.14998 (PMC7077598; doi:10.1111/jcmm.14998)
Supplement: Supplementary file 3 [file JCMM-24-3217-s003.docx]

**Supplementary Figure Legends**

**Figure S1.** The fundus photographs of three patients with PVR. (A) Right eye of patient P1, (B) right eye of patient P2 and (C) left eye of patient P3.

**Figure S2.** Autophagy inhibitors attenuate TGF-β2–induced EMT markers synthesis. (a, b) Western blot analysis of α-SMA, fibronectin and collagen IV in human primary RPE cells treated with TGF-β2 (10 ng/ml) in the absence or presence of either 3-MA (10 mM) or Baf-A1 (10 nM) for 24 h. GAPDH was used as loading control.
